# Nanoparticle-Based Systems for *T*_1_-Weighted Magnetic Resonance Imaging Contrast Agents

**DOI:** 10.3390/ijms140510591

**Published:** 2013-05-21

**Authors:** Derong Zhu, Fuyao Liu, Lina Ma, Dianjun Liu, Zhenxin Wang

**Affiliations:** 1 Department of Medicinal Chemistry and Pharmaceutical Analysis, Guangdong Medical College, Dongwan 523770, Guangdong, China; E-Mail: dr_zhu123@yahoo.com.cn; 2 State Key Laboratory of Electroanalytical Chemistry, Changchun Institute of Applied Chemistry, Chinese Academy of Sciences, Changchun 130022, Jilin, China; E-Mails: liufuyao@ciac.jl.cn (F.L.); liud@ciac.jl.cn (D.L.)

**Keywords:** *T_1_*-weighted magnetic resonance, molecular imaging, nanoparticles, contrast agents

## Abstract

Because magnetic resonance imaging (MRI) contrast agents play a vital role in diagnosing diseases, demand for new MRI contrast agents, with an enhanced sensitivity and advanced functionalities, is very high. During the past decade, various inorganic nanoparticles have been used as MRI contrast agents due to their unique properties, such as large surface area, easy surface functionalization, excellent contrasting effect, and other size-dependent properties. This review provides an overview of recent progress in the development of nanoparticle-based *T*_1_-weighted MRI contrast agents. The chemical synthesis of the nanoparticle-based contrast agents and their potential applications were discussed and summarized. In addition, the recent development in nanoparticle-based multimodal contrast agents including *T*_1_-weighted MRI/computed X-ray tomography (CT) and *T*_1_-weighted MRI/optical were also described, since nanoparticles may curtail the shortcomings of single mode contrast agents in diagnostic and clinical settings by synergistically incorporating functionality.

## 1. Introduction

Molecular-diagnostic imaging, a field at the intersection of molecular biology and *in vivo* imaging, has received considerable attention in the past decades due to its diagnostic and clinical promises [1–10]. Representative imaging platforms/techniques include computed X-ray tomography (CT), optical imaging, magnetic resonance imaging (MRI), positron emission tomography (PET), single-photon-emission computed tomography (SPECT), and ultrasound. These techniques hold promise because they allow real-time visualization of the cellular functions of living organisms and related molecular interactions, and, importantly, they are noninvasive. Of these methods, MRI is currently one of the most powerful diagnostic tools in medical science since it is able to acquire 3-D tomographical information in whole tissue samples, including human soft tissues, and whole animals, at high spatial and temporal resolution [1,5,11–15]. In addition, because MRI images are acquired without the use of ionizing radiation (X-ray/CT) or radiotracers (PET and SPECT), it has been the preferable imaging technique for the heart, brain, and nervous system [16–21]. Although tissue MRI is capable of revealing anatomic details in organs, it is difficult to differentiate normal and diseased cells due to small native relaxation time differences. In this context, imaging sensitivity can be enhanced through the use of MRI contrast agents [12,14,17].

MRI is based on the response of proton spin in the presence of an external magnetic field when triggered with a radio frequency (RF) pulse [11,22,23]. Under the influence of an external magnetic field, protons align in one direction. On application of the RF pulse, aligned protons are perturbed and subsequently relax to their original state. There are two independent relaxation processes: longitudinal (*T*_1_) and transverse (*T*_2_) relaxation, which are typically used to generate the MR images. Therefore, there are two classes of MRI contrast agents available, (1) *T*_1_-weighted contrast agents (e.g., gadolinium- (Gd^3+^) and manganese- (Mn^2+^) chelates) are paramagnetic in nature which increase the *T*_1_ relaxation time, resulting in bright contrast *T*_1_-weighted images; and (2) *T*_2_-weighted contrast agents are superparamagnetic materials (e.g., magnetite (Fe_3_O_4_) nanoparticles) which reduce *T_2_* relaxation times, giving rise to dark contrast *T*_2_-weighted images [11,22,23]. The efficiency of a contrast agent to reduce the *T*_1_ or *T*_2_ of water protons is referred to as relaxivity and defined by followed equation: 1/*T*_1,2_ = 1/*T*^0^_1,2_ + *r*_1,2_C. Where *1/T*_1,2_ is the observed relaxation rate in the presence of contrast agents, *1/T*^0^_1,2_ is the relaxation rate of pure water, C is the concentration of the contrast agents and *r*_1_ and *r*_2_ are the longitudinal and transverse relaxivities, respectively [11,21–23].

Recent advances in cross-disciplinary nanoscience and nanotechnology have led to further and rapid developments of novel nanomaterials as MRI contrast agents. Because of their importance in MRI, several reviews on synthesis and applications of nanomaterial-based contrast agents have been published elsewhere [8,9,14,16,17,24–40]. Compared to conventional MRI contrast agents, nanomaterial-based MRI contrast agents offer a number of advantages, (1) biostability and tunable biodistribution can be achieved by surface modification; (2) different degrees of biocompatibility and imaging properties can be adjusted by their chemical composition, shapes and sizes; (3) they can identify the desired target by specific conjugation with biological molecules interactions, such as antibodies, nucleic acids, and peptides; and (4) multimodal imaging can also be achieved using a combination of optical and magnetic properties of nanomaterials. The nanoparticle-based contrast agents were classified and discussed as *T*_1_-weighted MRI contrast agents, *T*_2_-weighted MRI contrast agents and multimodality imaging contrast agents. This review focuses on recent progresses in nanoparticle-based contrast agents for *T*_1_-weighted MRI and *T*_1_-weighted MR/optical (fluorescence and X-ray) multimodal imaging. The review highlights the design and fabrication procedures of nanoparticle-based contrast agents and their potential biomedical applications. In addition, we consider the following points: synthetic strategy for improving *r*_1_-relaxivity, design of the ligand shell to attain high colloidal stability and biocompatibility, and *in vivo* MR imaging of cancer. We refer interested readers to other review articles for *T_2_*-weighted MRI specific nanoparticulate systems (or in other word nanohybrids) [16,25,26–29,35,36].

## 2. General Description of *T*_1_*-*Weighted Contrast Agents

**Figure 1:**
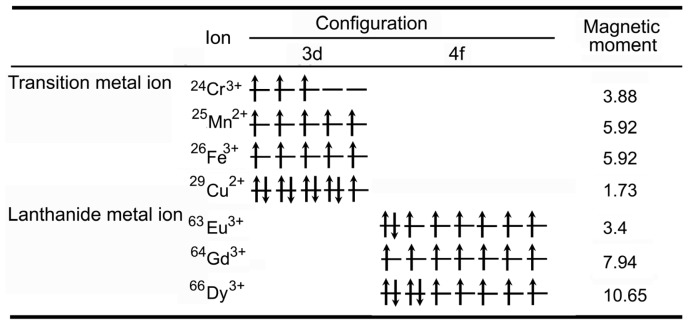
Electron configuration and magnetic moment of metal ions (adapted from Na *et al.* 2009 [29], Copyright 2009 WILEY-VCH Verlag GmbH & Co. KGaA, Weinheim and reproduced with permission).

*T*_1_ relaxation is the process of equilibration of the net magnetization (Mz) after the introduction of an RF pulse. This change of Mz is a consequence of the energy transfer between the proton spin system and the nearby matrix of molecules [11,21,22]. All biological systems are composed of various molecules and organisms which have different proton concentrations and different *T*_1_ relaxation times. The presence of paramagnetic ions (e.g., Gd^3+^ and Mn^2+^, as shown in Figure 1) near the tissue enhances its relaxation and shortens the *T*_1_ relaxation time [11,21–23,29]. Contrast agents with *T*_1_-weighted enhancing ability produce bright positive signal intensity in images and increase the conspicuousness of cells, facilitating easy tracking of cells in low-signal tissues [11,21–23]. Among those paramagnetic ions, Gd^3+^ is the most effective *T*_1_-weighted contrast agent for clinical use [17,29,41,42]. It is suggested that more than 10 million MRI studies are performed worldwide using Gd^3+^-based contrast agents each year [42]. Gd-chelates (e.g., Gd-diethylenetriaminepentaacetic acid (Gd-DTPA) and Gd-N,N′,N″,N‴-tetracarboxymethyl-1,4,7,10-tetraazacyclododecane (Gd-DOTA)) are normally used as *T*_1_-weighted MRI contrast agents. Despite their utility, these contrast agents suffer from poor sensitivity and rapid renal clearance, which severely limits the time window for MRI. Considerable efforts have been devoted to incorporate Gd^3+^ onto or into nanoparticles (e.g., hydrophilic macromolecule nanoparticles including dendrimers, dextran, and other hydrophilic polymers, liposomes, and inorganic nanoparticles (NPs)) [24,29,41–87]. This method can concentrate Gd^3+^ ion on/in the nanoparticles, resulting in the reduction of the toxicity of Gd^3+^, and enhancing the *T*_1_*-*weighted MR signal. It can also increase the cellular uptake of Gd^3+^ ions through size and shape tuning of the vehicle nanoparticles. Furthermore, biological functional groups can be conjugated on the nanoparticles surfaces for studying dynamic biomolecular phenomena through suitable biochemical reaction and signaling. Compared to Gd/Mn chelates, the nanoparticle-based *T*_1_-weighted MRI contrast agents have several advantages, including passive targeting properties, the prolonged imaging time, the enhancement of the contrast, and low toxicity [8,14,17,24,26,29,34]. Based on the compositions of nanoparticle-based *T*_1_-weighted MRI contrast agents, they can be classified as: (1) Gd-chelate grafted organic/inorganic nanoparticles, (2) gadolinium nanoparticles, (3) manganese-based nanoparticulate systems, and (4) dual (*T*_1_- and *T*_2_-) weighted MRI contrast agents.

## 3. Gd-Chelate Grafted Hydrophilic Macromolecule Nanoparticles

**Figure 2:**
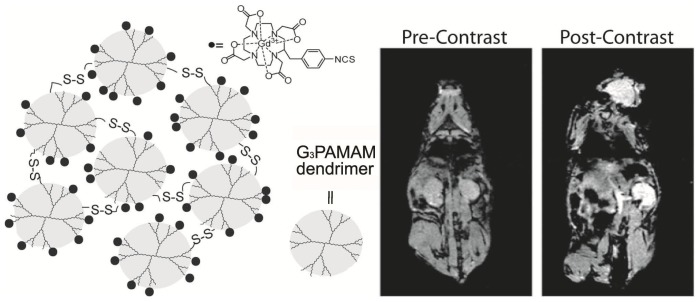
Schematic representation of Gd-conjugated polydisulfide dendrimer clusters and corresponding MR images of nu/nu nude mice before and after the tail vein injection of Gd-conjugated polydisulfide dendrimer clusters (adapted from Huang *et al.* 2012 [60], Copyright 2012 American Chemical Society and reproduced with permission).

**Figure 3:**
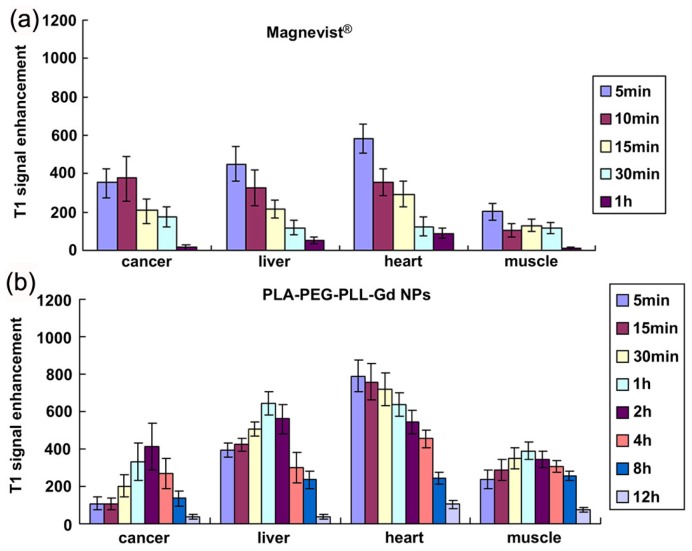

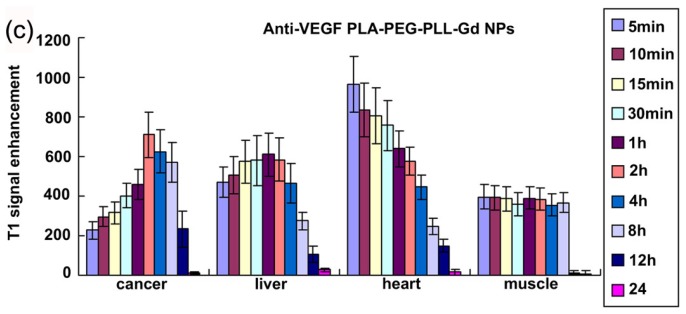
The results of the enhanced signal in different tissue *in vivo* [Magnevist^®^ (**a**), PLA-PEG-PLL NPs (**b**) and Anti-VEGF PLA-PEG-PLL-Gd NPs (**c**)] (adapted from Liu *et al.* 2011 [55], Copyright 2011 Elsevier Ltd. and reproduced with permission).

Polymeric Gd complexes, based on dextrans, polylysine derivatives, or dendrimers, as well as Gd-chelate grafted latex nanoparticles, liposomes, and micelles have been successfully employed for developing Gd-chelate grafted hydrophilic macromolecule nanoparticle contrast agents [42,50–61]. For instance, branched polyamidoamine (PAMAM) dendrimers are synthetic biocompatible macromolecules, processing multiple free amino groups on the surface. The physical, chemical, and biological characteristics of PAMAM dendrimers make this kind of molecules an ideal template for synthesis of Gd-chelate grafted nanoparticles [51,56,60]. Cheng and coauthors have provided a facile method for the synthesis of nanometer-sized dendrimer nanoclusters (5.8 nm in diameter) [56]. The dendrimer nanoclusters were labeled with Gd by reaction of the amine functional groups with the chelating agent DTPA dianhydride. The Gd-conjugated dendrimer nanoclusters have an *r*_1_ relaxivity value of 12.3 mM^−1^·s^−1^ per Gd^3+^ (1.41 T), which is much higher than that of Gd-DTPA (3.9 mM^−1^·s^−1^ per Gd^3+^) under same experimental condition. The ultrasensitive MR detection of various types of cancers may be possible by conjugating appropriate cancer-targeting ligands (e.g., folic acid) with the Gd-conjugated dendrimer nanoclusters. Huang and coauthors have recently reported gadolinium-conjugated PAMAM dendrimer nanoclusters as *T*_1_-weighted MRI contrast agents (as shown in Figure 2) [60]. In order to reduce the potential toxicity of PAMAM dendrimer clusters, individual Gd^3+^ labeled PAMAM dendrimers have been cross-linked to form larger nanoclusters through biodegradable polydisulfide linkages. These biodegradable polydisulfide dendrimer clusters inherit the high relaxivity (*T*_1_) of dendrimer clusters (the *r_1_* value is more than 11.7 mM^−1^·s^−1^ per Gd^3+^(1.41 T)) and retain an extended circulation time (circulation half-life > 1.6 h in mice). They are reduced to smaller degradation products while in circulation and undergo efficient renal excretion, reducing the possibility of long-term macromolecular particle retention. Importantly, the size of dendrimer nanoclusters can be easily controlled by adding an excess of maleimide to stop disulfide bond formation at different reaction time points. This strategy has provided a facile method for the synthesis of size-controllable and biodegradable dendrimer nanoclusters for clinical applications. Liu and coauthors designed a novel multifunctional polymeric nanoparticle contrast agent (Anti-VEGF PLA-PEG-PLL-Gd NP) simultaneously modified with Gd-DTPA and anti-vascular endothelial growth factor (VEGF) antibody to deliver Gd-DTPA to the tumor area and achieve the diagnosis of hepatocellular carcinoma (HCC) at an early stage [55]. The Anti-VEGF PLA-PEG-PLL-Gd NPs exhibited high *T*_1_ relaxivity [the *r*_1_ value is 18.394 mM^−1^·s^−1^ per Gd^3+^ (3.0 T)], good biocompatibility, and excellent selectivity towards tumor cells (as shown in Figure 3). The Anti-VEGF PLA-PEG-PLL-Gd NPs show great potential in the diagnosis of liver tumors at early stages.

## 4. Gd-Chelate Grafted Inorganic Nanoparticles

**Figure 4:**
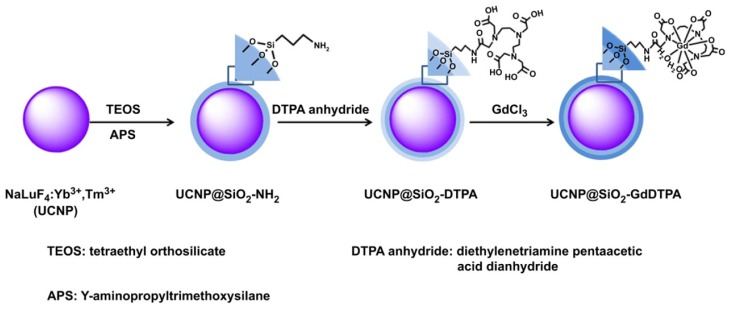
Schematic representation of the synthetic route of NaLuF_4_@SiO_2_-GdDTPA nanoparticles (UCNP@SiO_2_-GdDTPA) (adapted from Xia *et al.* 2012 [64], Copyright 2012 Elsevier Ltd. and reproduced with permission).

**Figure 5:**
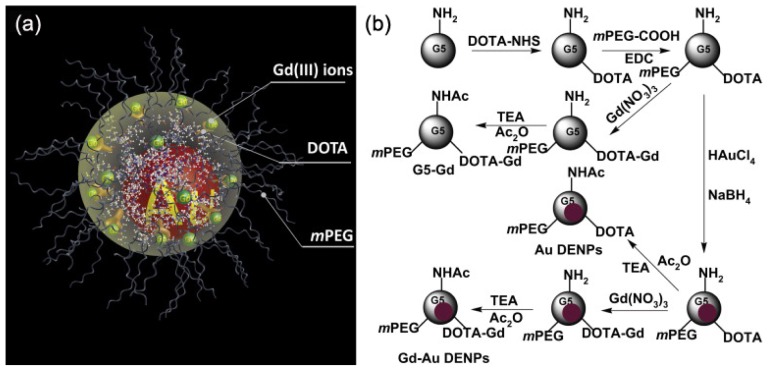
Schematic illustration of the designed nanostructure (**a**) and the synthesis procedure (**b**) of the Gd–Au DENPs. TEA and Ac_2_O represent triethylamine and acetic anhydride, respectively (adapted from Wen *et al.* 2013 [65], Copyright 2012 Elsevier Ltd. and reproduced with permission).

Gd-chelate grafted inorganic nanoparticles have recently attracted much attention because they could behave as contrast agents for *in vivo* multimodality imaging [62–69]. Multimodality imaging enables the providing of more complementary, effective, and accurate information about the physical, anatomical structure, and the physiological function for diagnosis and research. For instance, Gd-chelate (Gd- dithiolated derivatives of diethylenetriaminepentaacetic acid (DTDTPA)) functionalized gold nanoparticles (Au@DTDTPA-Gd nanoparticles) were prepared and applied as contrast agents for both *in vivo* CT and *T*_1_-weighted MRI by Alric and coauthors [62]. The nanoparticles were obtained by encapsulating gold nanoparticle cores within a multilayered organic shell which is composed of Gd-chelates bound to each other through disulfide bonds. Specific targeting of cancer at an early stage can be achieved by the covalent grafting of biotargeting groups on the organic multilayer of the Au@DTDTPA-Gd nanoparticles since each DTDTPA ligand possesses three COOH moieties as anchoring sites. The experimental results also demonstrated that the development of nanoparticles for targeted diagnosis and therapy can, therefore, be envisaged with the Au@DTDTPA-Gd nanoparticles. Xia and coauthors have designed and synthesized a kind of core@shell lanthanide-based nanoparticles, NaLuF_4_:Yb^3+^, Tm^3+^@SiO_2_-DTPA-Gd nanoparticles (UCNP@SiO_2_-GdDTPA) with NaLuF_4_:Yb^3+^, Tm^3+^ upconverting nanoparticles (UCNPs) as the core and SiO_2_ as the shell layer, and the Gd-DTPA as the surface ligand (as shown in Figure 4) [64]. The UCNP@SiO_2_-GdDTPA can be employed as a contrast agent for near-infrared to near-infrared (NIR-to-NIR) upconversion luminescence (UCL), CT and *T*_1_-weighted MR trimodality *in vivo* imaging. Gd^3+^ binding on the surface of nanoparticles makes the core@shell nanoparticles show high *r*_1_ relaxivity [6.35 mM^−1^·s^−1^ (0.5 T)] and suitable for *T*_1_-weighted MRI. The UCNP@SiO_2_-GdDTPA have been applied in the trimodal NIR-to-NIR UCL, CT, and *T_1_*-weighted MR molecular imaging for small animals, both *in vivo* and *in vitro*. Wen and coauthors have designed and synthesized multifunctional Gd-loaded Au DENPs (Gd–Au DENPs) [65]. In this case, amine-terminated generation 5 PAMAM dendrimers (G5·NH_2_), modified with Gd-chelator and polyethylene glycol (PEG) monomethyl ether, were used as templates to synthesize gold nanoparticles (Au NPs) within the dendrimer interior (Au DENPs) (as shown in Figure 5). The formed G5·NH_2_ with Gd-chelators were complexed with Gd^3+^, followed by complete acetylation of the remaining dendrimer terminal amines. The as-prepared Gd–Au DENPs are water soluble, colloidally stable, and non-cytotoxic in the given concentration range. The Gd–Au DENPs have an *r*_1_ relaxivity of 1.05 mm^−1^·s^−1^ per Gd^3+^ (3.0 T). The experimental result demonstrates that the Gd–Au DENPs enable to be used as dual mode (CT/MRI) contrast agents for *in vivo* imaging of some major organs of rats and mice. Taking into consideration of the unique structural characteristics of the dendrimers that can be further functionalized with various targeting ligands, it is expected that the developed Gd–Au DENPs may be used as a multifunctional nanoplatform for targeted CT/MR dual mode imaging of various biological systems, especially for diagnosis of cancer at early stage with high accuracy and high sensitivity.

## 5. Gadolinium Nanoparticles

Recently, inorganic nanoparticles containing Gd^3+^ [e.g., gadolinium oxide (Gd_2_O_3_), gadolinium fuoride (GdF_3_) and gadolinium phosphate (GdPO_4_)] have been investigated as *T*_1_-weighted MRI contrast agents [70–87]. Compare to Gd-chelate grafted nanoparticles, these nanoparticles have several distinct advantages: (1) ease of synthesis and functionalization, (2) the ability to carry large payloads of active magnetic centers, (3) decreased tumbling rates, which lead to increased relaxivity values. For example, most of these nanoparticles showed larger *r*_1_ values than those of the Gd-chelates, depending on their diameters. Ultrasmall Gd_2_O_3_ nanoparticles, with an average diameter of 1 nm, have been prepared by Park and coauthors [70]. Surface Gd^3+^ ions in the Gd_2_O_3_ nanoparticles cooperatively induce the longitudinal relaxation of the water proton. Thus, the ultrasmall Gd_2_O_3_ nanoparticles provided the *r*_1_ of 9.9 mm^−1^·s^−1^ (1.5 T), which is much larger than those of Gd-chelates. After injection with d-glucuronic acid coated ultrasmall Gd_2_O_3_ nanoparticles, high contrast *in vivo T*_1_-weighted MR images of the brain tumor of a rat were observed. Very recently, Liang and coauthors have developed a simple, cost effective, and easy to scale up strategy for synthesized poly-(acrylic acid)-coated, ultrasmall paramagnetic gadolinium hydrated carbonate nanoparticle (GHC-1) with small size (~2 nm in diameter) [78]. The GHC-1 exhibits a large longitudinal relaxivity of 34.8 mm^−1^·s^−1^ (0.55 T) while maintaining an *r_2_*/*r_1_* ratio as low as 1.17, making it effective as a *T*_1_-weighted MRI contrast agent. More importantly, the presence of carboxylic acid groups on the external surface of the nanoparticles is thought to be useful for conjugation of targeting molecules, such as antibodies and peptides, to the nanoparticles for specific tumor imaging.

Rare earth (RE) ions doped UCNPs have been of considerable interest in recent years due to their applications in biomedical imaging, solar cells, lasers, lighting, and display technologies [79–87]. Among upconverting nanomaterials, the Gd^3+^ contained UCNPs have shown excellent UCL, unique MR and strong X-ray attenuation. Therefore, the Gd^3+^ contained UCNPs can be employed as high performance contrast agents for UCL, MR and CT imaging. Anti-EGFR monoclonal antibody (mAb) conjugated NaGdF_4_ nanocrystals (NaGdF_4_-PEG-mAb) with narrow particle size distributions were synthesized by Hou and coauthors [86]. The experimental results revealed that the NaGdF_4_-PEG-mAb probes possessed satisfying tumor-specific targeting ability and strong MRI contrast enhancement effects. Liu and coauthors successfully synthesized a high-quality PEGylated Gd_2_O_3_:Yb^3+^, Er^3+^ nanorods (PEG-UCNPs) for *in vivo* UCL, *T*_1_-weighted MR, and CT multimodality imaging [87]. As an alternative to lanthanide-doped fluoride, the oxide-based nanoprobes feature superior properties, such as easy decomposition inside macrophage cells after reticuloendothelial system (RES) uptake and nearly total excretion from the mouse body.

## 6. Manganese-Based Nanoparticulate Systems

**Figure 6:**
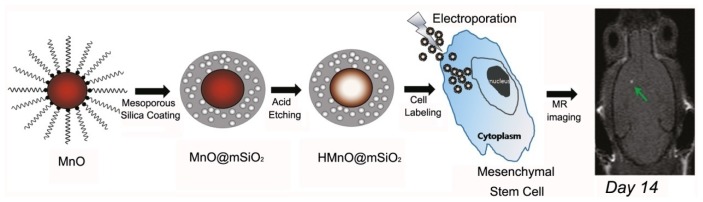
Schematic illustration of the synthesis of HMnO@mSiO_2_ nanoparticles and labeling of mesenchymal stem cells (MSCs) and *in vivo* magnetic resonance imaging (MRI) of MSCs transplanted mouse (adapted from Kim *et al.* 2011 [89], Copyright 2011 American Chemical Society and reproduced with permission).

**Figure 7:**
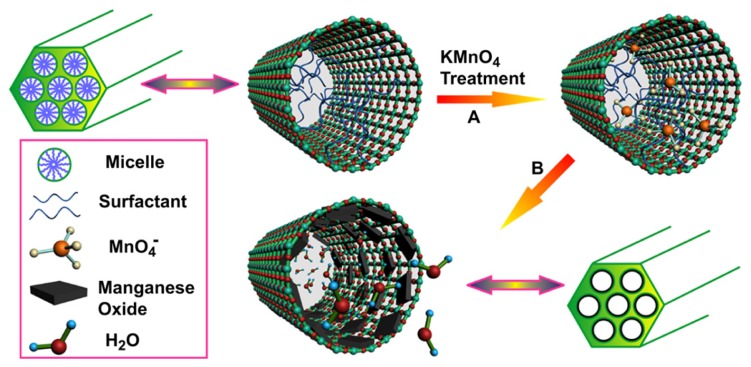
Schematic representation for the preparation of manganese oxide/mesoporous silica nanoparticles (MSNs)-based *T_1_*-weighted MRI contrast agents (adapted from Chen *et al.* 2012 [90], Copyright 2012 Elsevier Ltd. and reproduced with permission).

Mn^2+^ is another cation could be used as the MRI contrast agent since it has five unpaired electrons with long electronic relaxation time. However, it is very difficult to design and synthesize highly stable Mn^2+^ complexes with high sensitivities for clinical applications. This drawback can be overcome by building manganese-based nanoparticulate systems, such as MnO, Mn_3_O_4_, Mn_3_O_4_@SiO_2_, MnO@mesoporous SiO_2_, and even hollow MnO nanoparticles [88–94]. Many approaches have been developed to synthesize manganese oxide (MnO or Mn_3_O_4_) nanoparticles. One common route is to heat up Mn-oleate in a high boiling point solvent (e.g., 1-octadecene) to induce nucleation and particle growth [93]. This method allows the preparation of nanoparticles with accurate size control. The enhancement of the accessibility of the manganese paramagnetic centers to water molecules is the key issue to be addressed to design highly efficient manganese-based MRI contrast agents [88–94]. Kim and coauthors report on a novel design of MnO nanoparticles that have a ‘hollow’ MnO core structure and a coating consisting of mesoporous silica (HMnO@mSiO_2_) (as shown in Figure 6) [89]. These HMnO@mSiO_2_ nanoparticles showed a significantly higher *r_1_* relaxivity (0.99 mM^−1^·s^−1^ (11.7 T)) over other existing manganese oxide nanoparticle-based contrast agents. The porous coating, which enables water exchange across the shell, combined with the large surface area-to-volume ratio resulting from the novel structure increases water accessibility to the manganese core and consequently provides enhanced *T*_1_-weighted contrast. Both *in vivo* and *in vitro* experimental results demonstrated that the HMnO@mSiO_2_ nanoparticles have a great potential application of *T_1_*-weighted MRI cell tracking. Chen and coauthors have reported on a synthetic strategy of chemical oxidation/reduction reaction *in-situ* in mesopores, followed by hydrogen reduction, for the fabrication of non-toxic manganese oxide/mesoporous silica nanoparticle (MSN)-based *T_1_*-weighted MRI contrast agents with highly comparable imaging performance to commercial Gd-based agents (as shown in Figure 7) [90]. This strategy involves a “soft-templating” process to prepare MSNs, *in-situ* reduction of MnO^4 −^ by the “soft templates” in mesopores and heat treatment under reducing atmosphere, to disperse MnO nanoparticles within mesopores. This material system has two prominent advantages: (1) highly dispersed MnO nanoparticles in the penetrating mesopore system ensures the high water-accessibility to manganese paramagnetic centers, and (2) large surface area and pore volume of mesopores make take up a large amount of therapeutic agents within the pore system possible.

## 7. Dual (*T*_1_- and *T*_2_-) Weighted MRI Contrast Agents

Both *T*_1_-weighted and *T*_2_-weighted contrast agents have their own advantages and disadvantages. For example, Gd-based *T*_1_-weighted MRI contrast agents have excellent enhancement but they have risks of biological toxicity [95]. In terms of low toxicity, magnetic iron oxide nanoparticle-based *T*_2_-weighted MRI contrast agents have been proven to be one of the most promising contrast agents for clinical use because they are naturally found in human body. However, their negative contrast is often confused with a low-level MR signal arising from adjacent tissues such as bone or vasculature since the magnetite nanoparticles represent dark areas in MR images [96]. Therefore, it is highly desirable to prepare robust dual MRI contrast agents for overcoming the disadvantages of single modality contrast agents. The simultaneous use of positive and negative MR imaging that employs the same contrast agents will significantly improve detection accuracy [97–100]. Monodispersed water-soluble and biocompatible ultrasmall magnetic iron oxide nanoparticles (UMIONs, 3.3 ± 0.5 nm in diameter) generated from a high temperature coprecipitation route are successfully used as efficient positive and negative dual contrast agents of MRI by Li and coauthors [99]. The experimental results demonstrate the great potential of the UMIONs in dual contrast agents, especially as an alternative to Gd-based *T*_1_-weighted contrast agents, which have risks of inducing side effects in patients.

## 8. Conclusions and Outlook

Over last decade, extensive research has been conducted to develop nanoparticle-based *T*_1_-weighted contrast agents to overcome the drawbacks of clinic Gd chelate-based *T*_1_*-*weighted contrast agents and iron oxide nanoparticle-based negative *T*_2_-weighted contrast agents. These new MRI contrast agents including Gd chelate-grafted nanoparticle and Gd/Mn contained inorganic nanoparticles are expected to provide exquisite sensitivity and specificity in disease diagnosis and tracking biological processes such as cancer development and metastasis, cell evolution, and cell-to-cell interactions. In particular, MRI of nanoparticle-based *T*_1_-weighted contrast agents in the cardiovascular system has the potential to become a powerful technology in both basic science as well as clinical settings. In addition, multimodal imaging or simultaneous imaging and therapy can be achieved through the combinations of various nanomaterials.

Although many of these promises have been realized in the *in vitro* testing or preliminary animal studies, significant obstacles still exist in translating these results into clinical diagnosis. For instance, it is difficult to precisely trace the *in vivo* behavior of these nanoparticle-based *T*_1_-weighted contrast agents, such as accumulation, degradation, and clearance. To overcome these obstacles, several fundamental issues will have to be clearly addressed, including synthesis scale, human compatibility, long-term stability, targeting efficiency, and pharmacokinetics. Interdisciplinary collaborative research is needed to both optimize methods for synthesis of highly reproducible nanoparticle-based *T*_1_-weighted contrast agents and to gain a better understanding of correlation between the basic physicochemical properties of these nanoparticles and their *in vivo* biological behaviors. They should be surmountable in the near future because these challenges are not unique to MRI contrast agents.
